# Perioperative Management of Valvular Heart Disease in Patients Undergoing Non-Cardiac Surgery

**DOI:** 10.3390/jcm13113240

**Published:** 2024-05-30

**Authors:** Yashar Jalali, Monika Jalali, Ján Števlík

**Affiliations:** Faculty of Medicine, Comenius University in Bratislava, 5th Department of Internal Medicine, University Hospital Bratislava, Ružinov, Špitálska 24, 813 72 Bratislava, Slovakia, and Ružinovská 4810/6, 821 01 Bratislava, Slovakia; monika.jalali@outllok.com (M.J.); stevlik@ru.unb.sk (J.Š.)

**Keywords:** non-cardiac surgery, valvular heart disease, prosthetic valve, aortic stenosis, mitral stenosis, perioperative management

## Abstract

Postoperative cardiovascular complications (either in a hospital or within 30 days after the operation) are among the most common problems with non-cardiac surgeries (NCSs). Pre-existing cardiac comorbidities add significant risk to the development of such complications. Valvular heart disease (VHD), a rather frequent cardiac comorbidity (especially in the elderly population), can pose serious life-threatening peri-/postoperative complications. Being familiar with the recommended perioperative management of patients with VHD or an implanted prosthetic heart valve who are scheduled for NCS is of great importance in daily clinical practice. Although recently published guidelines by the European Society of Cardiology (ESC) and the American College of Cardiology and American Heart Association (ACC/AHA) for the management of VHD and perioperative management of patients undergoing NCS addresses the mentioned problem, a comprehensive review of the guidelines that provides an easy-to-use summary of the recommendations and their similarities and differences is missing in the published literature. In this review article, we summarize all of the relevant important information based on the latest data published in both guidelines needed for practical decision-making in the perioperative management of patients with VHD or after valvular repair (with prosthetic heart valve) who are scheduled for NCS.

## 1. Introduction

Annually, more than 280 million patients worldwide undergo NCS [1]. In the European Union alone, nearly 22 million major NCSs were performed in 2021 [1]. A comparison of the data shows that the number of NCSs has been rising by nearly 34% in recent years [2]. Every surgery carries its own peri-/postoperative risks/complications. The prevalence of such complications is determined by the comorbidities and clinical condition of the patient and by the urgency, magnitude, type, and duration of the surgical procedure.

Among the most frequent comorbidities in the modern era are cardiovascular complications [3]. A recent study from the USA demonstrated that nearly half of adults older than 45 years who are undergoing a major NCS will have at least two cardiovascular risk factors [4], which significantly increase their risk of major perioperative cardiovascular complications. In a study published by Spence et al. [5], from a cohort of 40,000 patients older than 45 years who underwent a major NCS, nearly 14% experienced a major cardiac complication 30 days after the operation.

One of the important cardiovascular risk factors in major operations is VHD. The European Society of Cardiology estimates that 13% of people aged 75 and older (in high-income countries) have VHD [6,7]. The prevalence of VHD markedly increases after age 65 [6]. Typically, severe VHD leads to the deterioration of heart function and may present with symptoms such as chest pain, dyspnoea, palpitations, and fainting [6]. However, in some cases, VHD, even in the severe stages, may involve no symptoms, and patients may not be aware they have it.

The magnitude of perioperative NCS risk in patients with VHD is highly variable and is dependent on the degree of severity of VHD and the type of NCS. The risk is particularly increased in patients with obstructive valve disease (such as mitral stenosis or aortic stenosis), where perioperative volume shift and/or arrhythmia can lead to cardiac decompensation [8,9]. Moreover, in patients with prosthetic heart valves, risk stratification is based on normal valve anatomy and valve function. In the case of mechanical heart valves, the importance of perioperative management centers around the correct evaluation of the anticoagulation regimen in regard to thrombus vs. bleeding risk.

In this paper, we present a risk stratification of patients with VHD based on the latest guidelines published by ACC/AHA and ESC and review possible approaches for perioperative management of such patients undergoing NCS.

## 2. Non-Cardiac Surgery Risk Stratification

Non-cardiac surgery-related risk is determined by the type and length of the operation. Surgical risk estimates broadly indicate a 30-day risk of cardiovascular (CV) death, myocardial infarction (MI), or stroke based on the proposed surgical intervention without considering the patient’s comorbidities. According to ESC, surgical risk can be categorized into low risk (<1% surgical risk), intermediate risk (1–5% surgical risk), and high risk (≥5% surgical risk), while the ACC/AHA categorizes surgical risk into low risk (<1% surgical risk) vs. elevated risk (≥1% surgical risk) [8,10]. The surgical risk stratification of NCS according to both guidelines is presented in Table 1.

## 3. General Preoperative Assessment

Clinical and echocardiographic evaluation is recommended for all patients with known or suspected VHD who are scheduled for elective intermediate or high-risk NCS [8,11]. Along with a thorough history and physical examination, a non-invasive transthoracic echocardiography (TTE, especially using 3D TTE) or four-dimensional computer tomography (4D CT) examinations are important to study the valve morphology, hemodynamics, left ventricular hypertrophy, systolic and diastolic functions of the left ventricle (LV), and other valvular complications [12]. Valvular pathologies (whether regurgitation or stenosis) are classified by ESC into three grades (mild, moderate, and severe) according to the severity of lesions. However, ACC/AHA categorizes valvular pathologies into four grades (A: at risk of development; B: progressive lesions (less than severe); C: asymptomatic severe; and D: symptomatic severe) according to the severity of lesions, hemodynamic consequences, and the patient’s functional status [11,13,14]. It should be mentioned that not all patients progress through all grades, and not all patients will meet all criteria (described in the guidelines) [11,13,14].

In cases of mild to moderate VHD diagnosed more than a year before the scheduled NCS, it is recommended to repeat echocardiography to re-evaluate possible progression [8,15].

Except for the grades of severity of valvular pathologies, etiologically, valvular regurgitations can be classified as primary (involving pathology of the valve apparatus, such as leaflets, chordae tendineae, and papillary muscles) or secondary (mostly involving pathologies of ventricles or atria). The severity of the valvular pathology, the primary or secondary characteristics of valvular regurgitation (in cases of mitral valve regurgitation), and the presence of symptoms can greatly impact the preoperative decision-making and management of patients prior to NCS.

In cases of severe VHD, it is very important to identify whether the symptoms are solely related to the underlying VHD (or other cardiovascular diseases) [12]. A symptomatic severe valvular disease profile, due to its elevated perioperative risk and mortality, in many cases, changes the approach to preoperative management and the timing of NCS.

Choosing the valvular repair procedure and valve type has to be done individually for each case according to societal guidelines and the decision of a multidisciplinary team of experts.

## 4. Aortic Valve Stenosis

The perioperative risk associated with aortic stenosis (AS) depends on the severity of stenosis, the presence of symptoms, and the coexistence of other cardiac or non-cardiac complications, mainly coronary artery disease (CAD) or chronic kidney disease (CKD).

The presence of CAD concomitant with AS in patients without a history or symptoms of CAD has been documented. Data from recent trials demonstrated a 30–40% prevalence of CAD in patients with the diagnosis of AS, among whom nearly one-third had no history of angina [16,17]. Development or worsening of myocardial ischemia in such patients has to be taken into consideration in the preoperative evaluation, operative plan and duration (if possible), and postoperative management.

Another important consideration prior to major NCS in patients with AS is renal function. Patients with CKD have a higher prevalence of AS, and AS severity correlates with CKD [12,18]. Pre-existing CKD is the most significant risk factor for the development of acute kidney injury (AKI), and the baseline estimated glomerular filtration rate (eGFR) is an important variable in preprocedural risk calculation [12,19].

Patients with mild to moderate aortic stenosis can undergo NCS regardless of their NCS risk profile [8,11].

### 4.1. Symptomatic Severe Aortic Stenosis

To evaluate the severity of aortic stenosis, a thorough clinical examination, including electrocardiography, transthoracic echocardiography, transoesophageal echocardiography (when applicable), and laboratory biomarkers, is recommended [8]. Symptoms, if present, have to be evaluated in accordance with optimizing the recommended medical treatment, and the diagnosis of symptomatic AS must be made after thorough clinical examination when symptoms persist despite optimized treatment [8].

Symptomatic severe AS poses a great risk for postoperative cardiac complications such as myocardial infarction and heart failure and is a predictor of 30-day mortality after NCS [8]. Patients with severe AS are likely to have an increased risk of a major advance cardiac event and intraoperative mortality [12,20]. The mechanism by which this occurs is thought to involve hypotension and tachycardia from anesthetic agents and surgical stress, leading to injurious hemodynamics. An unfavorable hemodynamic state can lead to decreased coronary perfusion, myocardial injury, the development of arrhythmias or ischemia, or death [12]. Despite published data suggesting reduced morbidity and mortality (in-hospital and 30-day) for patients with aortic valve replacement (AVR) before elective intermediate or high-risk NCS [21], the decision regarding the timing of AVR in relation to NCS should be made according to the baseline risk profile and associated risk of NCS [8,22].

The recommendations of the ACC/AHA and ESC guidelines regarding the approach for patients with symptomatic severe AS undergoing low-, moderate-, or high-risk NCS considering the risk of AVR are summarized as follows:

- For patients with symptomatic severe aortic stenosis for whom AVR surgery does not pose a high operative risk and NCS is not time-sensitive (regardless of the NCS risk profile), SAVR or TAVI is recommended before NCS [8,10,11];
- For patients with symptomatic severe aortic stenosis for whom NCS is intermediate to high-risk and is time-sensitive and AVR poses a high operative risk, TAVI is preferred over SAVR before NCS [8,10,11];
- For patients with symptomatic severe aortic stenosis for whom NCS is intermediate to high-risk and is time-sensitive and neither TAVI nor SAVR is feasible, balloon aortic valvuloplasty (BAV) may be considered before NCS as bridging therapy until planned definitive aortic valve repairment [8,10,11].

### 4.2. Severe Asymptomatic Aortic Stenosis

Aortic valve intervention in asymptomatic patients with severe AS and normal left ventricular ejection fraction (LVEF) undergoing low- to intermediate-risk NCS is not needed if the NCS is not associated with a large volume shift [8]. Appropriate intraoperative and postoperative hemodynamic monitoring is recommended [8,10,11].

In asymptomatic patients with severe AS undergoing high-risk NCS, the decision to perform AVR has to be made individually based on the patient’s clinical presentation and a comparison of the risk profile of AVR vs. NCS [8,10,11].

## 5. Aortic Valve Regurgitation

**Figure 1:**
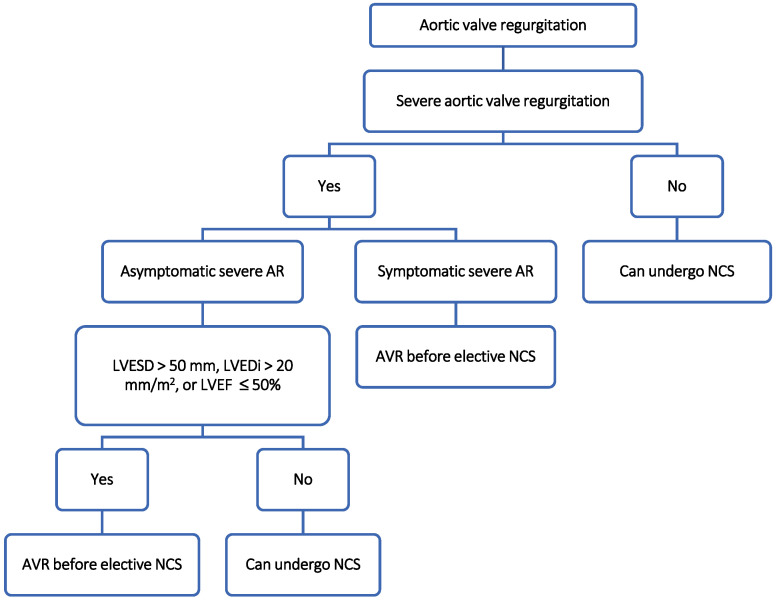
Suggested management algorithm of patients with aortic regurgitation undergoing non-cardiac surgery [8,10,11]. Data used in the production of the figure are derived from the ESC and/or ACC/AHA guidelines cited above. Data/algorithm presented are interpretation of authors from data available on cited guidelines and/or academic published data on the topic and should not be used instead. For more information study of corresponding guideline is always recommended. AR: aortic regurgitation; AVR: aortic valve repair; LVEF: left ventricular ejection fraction; LVESD: left ventricular end-systolic diameter; LVESDi: left ventricular end-systolic diameter index; NCS: non-cardiac surgery.

The approach for patients with aortic valve regurgitation (AR) is quite similar and rather straightforward in both ACC/AHA and ESC guidelines:

- Patients with mild to moderate AR can undergo NCS regardless of the NCS risk profile [8,11];
- Patients with severe symptomatic AR or with severe asymptomatic AR with a left ventricular end-systolic diameter (LVESD) of >50 mm, an LVESD index (LVESD/body surface area) of > 25 mm/m^2^ (patients with small body size), or a resting LVEF of ≤ 50% (<55% according to ACC/AHA guidelines), AVR is recommended before elective intermediate- to high-risk NCS (Figure 1) [8,11].

Aortic valve surgery may be considered for asymptomatic patients with LVEF > 55% and LVESDi > 20 mm/m^2^ according to ESC and for asymptomatic patients with LVEF > 55% with a low risk of AVR and documented signs of worsening LFEV or signs of an increased LV end-diastolic diameter of >65 mm (on at least three consecutive echocardiography exams) according to ACC/AHA [8,11]. In this sense, AVR could be considered for patients who are undergoing elective NCS with elevated risk for whom valvular repair does not pose a high risk.

## 6. Mitral Valve Stenosis

Rheumatic heart disease is still the primary cause of mitral stenosis (MS) in most developing countries. MS is also attributed to primary age-related degenerative valve changes and congenital mitral valve abnormalities.

Echocardiography plays a major role in the diagnosis of MS, quantitation of stenosis severity, assessment of hemodynamic consequences, and analysis of valve anatomy [13]. ACC/AHA and ESC have different approaches to the echocardiographic diagnosis of MS with regard to the methodology used [13,23]. In the ESC guidelines, the severity of MS is determined based on mitral valve area (MVA), trans-mitral flow mean gradient, and systolic pulmonary arterial pressure (sPAP). In the ACC/AHA guidelines, the severity of MS is determined using MVA and diastolic pressure half-time [11].

In the ACC/AHA guidelines, the need for intervention is categorized based on a rheumatic or non-rheumatic etiology of MS. For patients with non-rheumatic calcific MS (most frequent cases in the European Union and developed countries), the guidelines recommend considering intervention after an extensive evaluation of the risks vs. benefits of operating and only in severely symptomatic patients with severe stenosis [11].

In the new ESC guidelines, however, the approach to the management of MS does not consider etiology and is based on the severity of valve pathology [8]. For a general approach and easier clinical interpretation, we selected the ESC guidelines for the perioperative management of MS in patients undergoing NCS.

**Figure 2:**
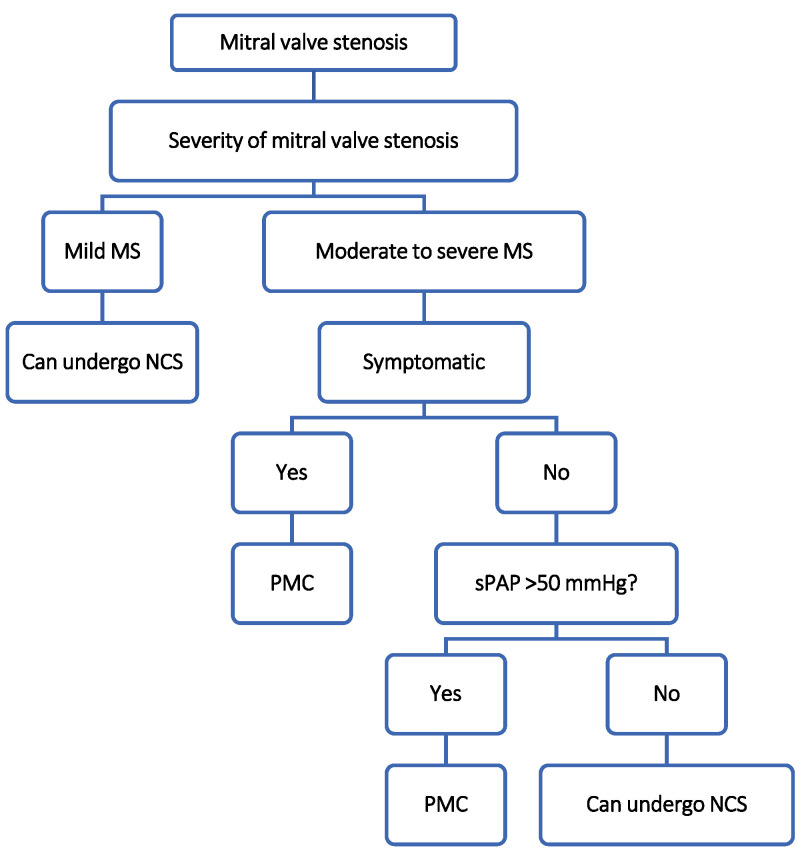
Suggested management algorithm of patients with mitral stenosis undergoing non-cardiac surgery [8]. Data used in the production of the figure are derived from the ESC and/or ACC/AHA guidelines cited above. Data/algorithm presented are interpretation of authors from data available on cited guidelines and/or academic published data on the topic and should not be used instead. For more information study of corresponding guideline is always recommended. MS, mitral stenosis; NCS, non-cardiac surgery; PMC, percutaneous mitral commissurotomy; sPAP, systolic pulmonary arterial pressure.

- According to ESC, patients with mild MS and asymptomatic patients with moderate to severe MS with sPAP < 50 mmHg can undergo NCS regardless of the risk profile (Figure 2) [8,10].
- For patients with asymptomatic moderate to severe MS with sPAP > 50 mmHg and patients with symptomatic moderate to severe MS (with increased risk of the perioperative cardiovascular event) who are undergoing high-risk NCS, a percutaneous mitral commissurotomy (PMC) should be considered before NCS [8]. For patients with severe symptomatic MS or severe asymptomatic MS with sPAP > 50 mmHg who are ineligible for PMC, high-risk NCS should be performed only if necessary (Figure 2) [8,10].

Since the majority of patients with MS (even in moderate to severe stages) will not undergo preoperative valve repair (Figure 2), it is very important to be familiar with possible perioperative complications and their management.

A chronic underfilled state of LV in long-term moderate to severe MS could induce LV muscle mass atrophy and LV dysfunction [10]. Tachycardia due to decreased diastolic LV filling time is poorly tolerated [23]. Since in patients with severe MS, the heart is in a low fixed cardiac output (CO) state, precautions need to be taken to avoid any increase in heart rate during the perioperative period and the induction of anesthesia [23]. Atrial fibrillation because of a dilated left atrium (LA) and a high risk of thrombus formation due to low-velocity blood flow in LA are other common complications of MS [10]. Due to the loss of atrial contraction during AF causing an elevation in the pressure gradient across the valve and increased pulmonary artery pressure, it is important to preserve sinus rhythm in these patients. That can be achieved with the perioperative use of digoxin in patients who are already on this medication or the use of short-acting beta-blockers, calcium channel blockers, or amiodarone, or electrical cardioversion in hemodynamically unstable patients (Table 2) [23].

Right ventricular (RV) dysfunction is the second major concern in patients with MS [8]. Chronic pulmonary vasoconstriction secondary to long-standing elevated LV pressure can lead to compensatory RV hypertrophy following chronic pulmonary hypertension, which can cause RV dilatation and consequent RV failure [23]. Hence, fluid should be administered cautiously. Over-transfusion in patients with MS can precipitate sudden pulmonary edema in an already elevated chronic pulmonary hypertensive vasculature [23]. In MS, afterload reduction does not help augment forward flow because stroke volume is determined by the mitral valve orifice area and the diastolic filling interval [23].

Due to deep concern for RV dysfunction, the medical team should take all measures to avoid increased sPAP. Hence, oversedation should be avoided to prevent hypoventilation and hypercapnia [23].

In general, the perioperative management of patients with MS should be focused on controlling heart rate and ventricular preload and managing RV–LV contractile function and coexisting pulmonary hypertension [23].

## 7. Mitral Valve Regurgitation

In the management of patients with mitral valve regurgitation (MR), echocardiography is essential not only for determining the severity and hemodynamic consequences of MR but also for its etiology. In the echocardiographic examination, it is important to differentiate between primary or degenerative MR and secondary MR.

The risk of NCS in these patients depends on the etiology, severity, level of hemodynamics and compensation, and NCS risk profile [24]. Predictors of increased cardiovascular risk in patients with moderate to severe MR undergoing NCS include AF, ischemic MR, and comorbidities such as, e.g., diabetes mellitus [24]. In patients with moderate to severe MR, the cardiac risk of NCS is also affected by other heart comorbidities, such as CAD, LV dysfunction, and LV dilatation [24]. Hence, besides determining the severity of MR, it is important to define LV function and LV size for the risk stratification of patients undergoing NCS.

**Figure 3:**
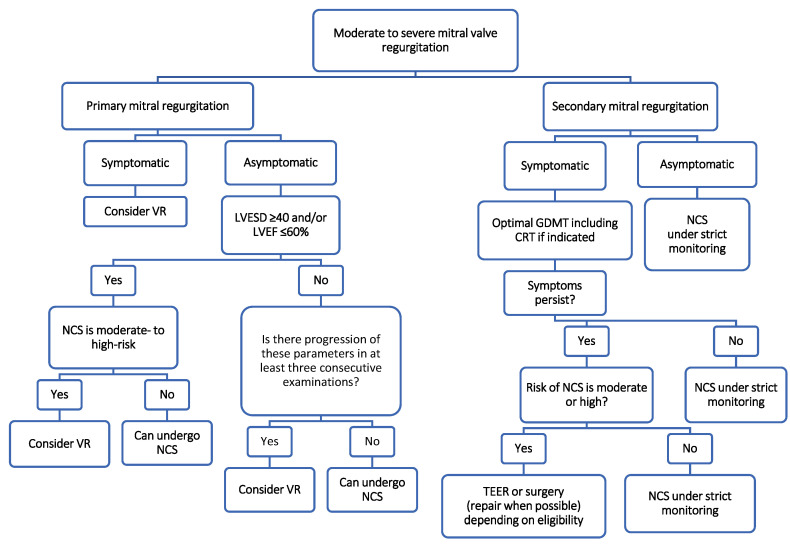
Suggested management algorithm of patients with aortic regurgitation undergoing non-cardiac surgery [8,11]. Data used in the production of the figure are derived from the ESC and/or ACC/AHA guidelines cited above. Data/algorithm presented are interpretation of authors from data available on cited guidelines and/or academic published data on the topic and should not be used instead. For more information study of corresponding guideline is always recommended. CRT, cardiac resynchronization treatment; GDMT, guideline-directed medical therapy; LVEF, left ventricular ejection fraction; LVESD, left ventricular end-systolic diameter; NCS, non-cardiac surgery; TEER, transcatheter edge-to-edge repair; VR, valvular repair.

Based on the ESC guidelines, the following recommendations are made:

- For patients with symptomatic severe primary MR or asymptomatic severe primary MR with LV dysfunction (defined as LVEF ≤ 60%) and/or LV dilatation (defined as LVESD ≥ 40 mm), valve intervention (surgical or transcatheter) should be considered prior to intermediate- or high-risk NCS if time allows (Figure 3) [8];
- For patients with severe secondary MR who remain symptomatic despite guideline-directed medical therapy (GDMT), including cardiac resynchronization therapy (CRT), valve intervention (transcatheter or surgical) should be considered before NCS when there is an acceptable procedural risk (Figure 3) [8].

Based on the above points, generally, ESC recommends valvular repair for patients with primary severe symptomatic MR or primary severe asymptomatic MR with LV dysfunction and for a very selected group of patients with secondary severe symptomatic MR (who are not responding to optimal treatment) for whom NCS is elective and poses moderate to high operative risk (Figure 3).

The ACC/AHA guidelines for the management of patients with MR are quite similar to the ESC guidelines, with one important difference [11].

The ACC/AHA approach is that any adult patient who meets the criteria for valvular intervention to address MR should undergo valve repair before elective NCS due to the reduced perioperative risk [11]. In this sense, the ACC/AHA guidelines recommend valvular intervention for asymptomatic patients with severe primary MR even before LVEF falls below 60% or LVESD rises above 40 mm once the patient meets the criteria for acceptable surgical risk and if the worsening of those parameters is detected in three serial imaging studies [11].

The rationale for the more aggressive AAC/AHA approach is that by the time the EF falls below 60% and LVESD increases above 40 mm, the LV function is already abnormal, and the patient is likely to enter the transitional phase of chronic primary MR [11]. Hence, the ACC/AHA guidelines emphasize that even mildly reduced EF (and/or its progression) in patients with severe MR represents a significant LV compromise [11].

The newer options for mitral valve repair, such as transcatheter edge-to-edge repair (TEER), have expanded the range of options available in recent years, especially for patients who are not eligible for open surgery [11,25]. Recent data from a carefully selected group of patients suggest that mitral TEER could improve longevity with fewer symptoms [11]. In light of these findings, the 2020 ACC/AHA guidelines increased the strength of their recommendations with regard to TEER [11,25].

The guidelines state that mitral valve TEER is reasonable for patients with grade C primary asymptomatic severe MR and LV impairment, grade D primary symptomatic severe MR, or severe secondary MR with a reduced EF of 20–50%, LVESD ≤ 70 mm, and sPAP ≤ 70 mmHg despite optimal medical treatment, who have high surgical risk, favorable anatomy for TEER, and a life expectancy of >1 year.

The 2021 ESC guideline mentions considering TEER for patients with primary symptomatic MR ”who fulfil the echocardiographic criteria of eligibility, are judged inoperable or at high surgical risk by heart team” (level of recommendation IIb and level of evidence B) [6] or for patients with secondary symptomatic MR who stay symptomatic despite optimal treatment “who are fulfilling criteria suggesting an increase chance of responding to TEER” (level of recommendation IIb level of evidence C) and are undergoing high-risk NCS (Figure 3) [6].

Age, gender differences, and comorbidities have been shown to play an important role in the determination of outcomes of valvular operation and the selection of type of valvular repair [26]. Differences in anatomical structures, pathophysiology, and different incidences of various diseases between males and females are important defining factors for such gender-related postoperative outcomes [26]. As an example, it has been shown that left ventricular reversal restructure after transcatheter mitral valve repair is an important prognostic factor in both genders [27]. Since fewer females reach the recommended surgical criteria of ventricular enlargement in MR, logically, worse postoperational outcomes may be expected [27]. A recently published meta-analysis on gender-related differences in the outcomes of transcatheter mitral valve repair demonstrated that males who underwent the procedure had worse preoperative comorbidities in comparison to females, yet they had better postoperative objective heart function at one year [28]. These differences in gender-related postoperative outcomes might be one of the reasons why, despite the predominance of degenerative mitral valve prolapse in females, more males are undergoing valve repair operations.

These gender-related differences are not only limited to mitral valve repair procedures but also aortic valve operations. For example, in a study published by Caponcello et al., gender-related differences in surgical versus transcatheter aortic valve replacement were examined [29]. In their study, no gender-associated differences were found in TAVR in terms of procedural success or postoperational mortality between males and females [29]. In contrast, SAVR was associated with a higher risk of short-term mortality in females due to older age and a higher number of comorbidities in their study cohort [29]. Hence, these factors have to be taken into consideration not only for the stratification of patients before NCS but also for the determination of the risk of the valvular repair procedure and the type of operation selected.

Although the available data are still limited, most patients with good functional status and preserved LV function can tolerate general anesthesia despite severe MR. Thus, elective NCS can be performed in adults with asymptomatic severe MR and preserved LV systolic function with less than severe sPAP (<50 mmHg) [24].

Since the majority of patients with MR undergo NCS without valvular repair (VR), the perioperative management of these patients is very important. In such patients, preload should be maintained or reduced to preserve intravascular volume and avoid fluid overload. Due to the increase in LV volume and compliance, excessive systemic afterload should be avoided [10]. A markedly increased blood pressure can increase MR and result in diminishing forward flow, and thus should be avoided [10]. Diuretics and afterload reduction should be carefully titrated to optimize perioperative hemodynamic status [10].

## 8. Prosthetic Valves

Patients who have undergone the correction of VHD and have a prosthetic valve can undergo NCS without any further contraindications if there is no evidence of valve dysfunction [8]. In this sense, it is recommended to perform a complete echocardiographic examination before elective NCS to evaluate the function of the valve. Prosthetic valve dysfunction, clotting, dislocation, leaking, stenosis, etc., generally involve serious complications, and its management, regardless of NCS, should be approached based on societal guidelines [30].

### Modification of Anticoagulation Therapy for Patients with Prosthetic Valves

In today’s practice, the main problem regarding prosthetic valves is the need to modify the anticoagulation regimen in the perioperative period [8]. The latest ESC and ACC/AHA guidelines indicate that lifetime anticoagulation treatment with vitamin K antagonist (VKA) is required for patients with mechanical heart valves (MHVs) [6,11,31]. Direct oral anticoagulants (DOACs) currently have no role in anticoagulation therapy for MHVs [6,11,32]. In current practice, treatment with VKA starts on the first post (valvular) operative day in combination with bridging therapy (either unfractionated heparin (UFH) or off-label low-molecular-weight heparin (LMWH) in therapeutic doses) until the targeted therapeutic international normalized ratio (INR) is achieved, then, bridging therapy is discontinued once a stable therapeutic INR is reached for more than 24 h [6,33,34].

Strict use of VKA is also recommended for patients with moderate to severe MS and atrial fibrillation (AF), with a targeted INR of 2–3 [6,8,35].

A narrow therapeutic window, highly variable individual dose response, and various drug-food interactions in the treatment with VKA (such as warfarin) require frequent blood testing and the adjustment of the drug’s dosage in order to achieve targeted INR [36]. Targeted values of INR should provide a balance between the risk of thromboembolic events and bleeding [37]. Several studies (from cohorts of patients with atrial fibrillation or venous thromboembolism) have demonstrated that the time spent in the therapeutic range (TTR) as a measure of the quality of anticoagulation with VKA is often poorly sustained, causing the development of adverse outcomes [38,39]. Such data from a cohort of patients with MHV, however, are limited. In patients with MHVs, targeted INR ranges from 2.5 to 4 depending on prosthetic thrombogenicity and the patient’s related risk factors (such as previous thromboembolism, AF, concomitant mitral stenosis of any degree, and LVEF < 35%) [6]. The adjustment of a VKA dose to achieve therapeutic ranges for a given MHV in an individual (in relation to the patient’s comorbidities, age, and gender, such as pregnant women) should be performed frequently enough to ensure the sustainability of the TTR and minimization of the adverse effect. Hence, it is important to be familiar with the targeted INR for any given type of implanted MHV for the management of perioperative anticoagulation.

Bleeding risk increases exponentially with INR > 4.5 [6]. Based on the 2021 ESC and European Association of Cardiothoracic Surgery (EACTS) guidelines and the 2020 ACC/AHA guidelines for the management of valvular heart disease, the modification of anticoagulation therapy for patients with MHVs in different scenarios can be carried out as follows:

- In the case of major and/or life-threatening bleeding, VKA overdose or need for urgent surgical operation (of any type) in patients with MHV, it is recommended to discontinue VKA and administer 10 mg of vitamin K by slow infusion (repeat every 12 h if necessary) [6,40]. Until the anticoagulation effect is reversed, fresh frozen plasma (FFP) and/or prothrombin complex (PCC) should be initiated according to body weight and pre-treatment INR [6]. Treatment efficacy should be evaluated at 30 min with the control of INR and then every 46 h until normalization [6]. Restarting anticoagulation therapy should be discussed according to the location of bleeding, intervention performed to stop bleeding, and surgical operation [6];
- In the absence of bleeding, for patients with MHV and overdose of VKA (INR > 10), oral vitamin K (2.5–5 mg) can be administered, and VKA should be stopped, with the monitoring of INR once daily for 2 weeks [6];
- In the absence of bleeding, for patients with MHV and overdose of VKA (INR 4.5–10), current evidence does not indicate any difference in bleeding events in patients administered vitamin K vs. placebo. Hence, it is recommended to stop VKA in these patients and follow INR. A small dose of oral vitamin K (1–2 mg) can be considered on an individual basis [6];
- In the absence of bleeding, for patients with MHV and INR < 4.5 but higher than the targeted value, it is recommended to down-titrate or skip one or more doses of VKA [8].

In all patients with MHVs, VKA must be restarted once the INR reaches the therapeutic range (in the absence of active bleeding) [6].

In patients with MHVs on VKA treatment undergoing elective NCS, is not recommended to interrupt oral anticoagulation treatment if the NCS is minor (e.g., dental, cataract, skin incisions) in which bleeding is minimal and can be easily controlled [11,34].

In patients with MHVs on VKA treatment undergoing elective major NCS, it is recommended to temporarily interrupt the oral anticoagulation treatment with therapeutic bridging (with either UFH or LMWH). It is recommended to perform daily INR measurements (during bridging therapy) aiming to achieve a target value of INR < 1.5 before operation [6].

The major difference between the ESC and ACC/AHA guidelines in the pre-perioperative modification of anticoagulation treatment concerns a specific group of patients with implanted bileaflet mechanical AVs and no other risk factor for thromboembolism who are undergoing an invasive procedure. The ACC/AHA guidelines recommend only interrupting VKA treatment 2 to 4 days before procedure without the need for bridging therapy, whereas the ESC guidelines do not provide any specific recommendation about this subgroup of patients.

Algorithm of modification of anticoagulation therapy and bridging strategies for patients undergoing major NCS with MHV on VKA from both guides indicates [6,11]:

Before intervention:

- To stop VKA about 5 days before intervention. On the next day (4 days before intervention) start UFH or LMWH as bridging therapy.
- Stop LMWH (as bridging therapy) 24 h before intervention, in case of UFH it can be stopped 6 h before intervention.

After intervention:

- Twelves to twenty-four hours (depending on operation type) after intervention start the UFH.
- On the first day after intervention start VKA therapy concomitant to UFH. The UFH can be switch to LMWH (if needed) in about 2 days after intervention.
- Stop concomitant UFH or LMWH therapy once INR more than 2 reached in patients with aortic valve prosthetic (AVP), and once INR more than 2.5 reached in patients with mitral valve prosthetic (MVP).
- In case of patients undergoing urgent NCS with biological heart valve (BHV) on DOAC or patients on lifelong DOAC, anticoagulation treatment can be stopped 2 days before intervention.
- After intervention UFH or LMWH can be added (without concomitant DOAC) in same time intervals as presented in VKA.
- Treatment with DOAC can be resume 3 days after intervention and UFH/LMWH can be stopped at the same time.

Fondaparinux should not be used routinely for bridging therapy, but it may have a role in therapy for patients with a history of heparin-induced thrombocytopenia (HIT) [6].

The recommendation in both guidelines for anticoagulation treatment for patients implanted with a BHV with no other indications for anticoagulation includes oral anticoagulation with VKA for 3 months for mitral BHV or mitral surgical valve repair and oral VKA or low-dose aspirin for 3 months for aortic BHV [8,11] or single low-dose aspirin therapy for 3 months after aortic surgical valve repair.

Lifelong single low-dose aspirin therapy is indicated for patients with bioprosthetic TAVI [8,11].

Although there are slight differences in the duration of treatment and/or the combination of antiplatelet medications (for patients with specific types of valves) between the ACC/AHA and ESC guidelines, for the purpose of simplification, we present the main recommendations accepted by both associations.

With regard to anticoagulation treatment for patients with BHV and AF, both guidelines recommend the use of DOACs for a period of 3 months over VKA and then as lifelong therapy [8,11].

Hence, elective NCS should be scheduled a minimum of 3 months after valvular bioprosthetic therapy. For urgent NCS within 3 months of valvular repair or for patients on lifetime anticoagulation therapy, the perioperative modification of anticoagulation treatment with bridging therapy is recommended.

Despite the fact that the routine monitoring of DOAC plasma levels may be deemed unnecessary due to their wide therapeutic index in the general population, recent data suggest the existence of clinical situations (like obesity, drug interaction, impaired hepatic or renal function, extreme age, etc.) where out-of-range DOAC concentrations (too high or too low) might need laboratory testing to ensure the drug’s treatment effectiveness [41,42]. Too low a drug concentration will not exert the expected anticoagulation effect and increase the risk of thromboembolism, while too high a drug concentration will increase the risk of bleeding [43]. Hence, recent efforts have been directed toward the evaluation of therapeutic drug monitoring (TDM) of DOACs. TDM assumes the existence of a relationship between the drug’s serum concentration and its effectiveness [43]. This concentration will be further related to a therapeutic range associated with the lowest risk of adverse effects and the highest possibility of the desired treatment outcome [44]. Due to limited data available to support dose adjustment, lack of formal implemented guidelines for the control of drug levels and the correlation of such data with thromboembolism or bleeding, the topic of TDM for DOACs remains (for the time being) a domain in need of further developments [45]. However, one may need to take into consideration possible undesired outcomes in such clinical scenarios, especially for patients in need of lifelong DOAC treatment.

## 9. Conclusions

Valvular heart disease in its severe stages is associated with a high risk of perioperative complications and a high mortality rate. A thorough examination and evaluation of VHD, an evaluation of prosthetic valve function, and an assessment of concomitant cardiac and relative non-cardiac disease should be performed before any elective NCS. Identifying whether symptoms are solely related to underlying VHD is an important part of the patient assessment, as in some cases, the presence of symptoms related to severe VHD alone can change the preoperative approach and management. Symptomatic patients, in general, have an increased risk of postoperative complications and mortality if they are undergoing moderate- to high-risk NCS. Where indicated, and particularly for symptomatic patients, valve repair should be considered prior to planned NCS. The type of valve repair procedure (transcatheter, open surgery, etc.) should be decided by a multidisciplinary team of experts, considering the risk of the valve repair operation, valvular characteristics, vascular anatomy, urgency, and risk of NCS of each individual case. Anticoagulation therapy should be strictly modified before elective NCS, especially for patients with MHV, due to the high risk of thromboembolic events vs. major life-threatening perioperative bleeding. Routine societal guidelines should be followed in the management of VHD regardless of the need for NCS.

## Figures and Tables

**Table 1:** Risk stratification of non-cardiac surgery according to ESC and ACC/AHA [8,11].

Low-Risk NCS (ACC/AHA)	Elevated-Risk NCS (ACC/AHA)	
Low-Risk NCS (ESC)	Intermediate-Risk NCS (ESC)	High-Risk NCS (ESC)
-Plastic surgeries; -Ophthalmic surgeries; -Thyroid surgeries; -Breast surgeries; -Dental surgeries; -Minor gynecological, orthopedic, and urological surgeries.	-Head and neck surg.; -Intraperitoneal surg.; -Kidney transplants; -Endovascular aortic aneurysm repair; -Carotid surgeries; -Major gynecological, orthopedic, urological, and neurological surg.	-Lung or liver transplant; -Hepatic resection; -Intrathoracic surgeries; -Peripheral vascular surg.; -Duodenal and pancreatic surgeries; -Aortic surgery; -Adrenal resection; -Perforated bowel surg.

ACC/AHA: American College of Cardiology and American Heart Association; ESC: European Society of Cardiology; Surg.: surgeries.

**Table 2:** Perioperative rhythm control management for patients with MS [23].

Medication	Dose
Digoxin	Loading dose of 0.25 mg IV over 15 min followed by 0.1 mg every hour until a response occurs or total dose of 0.5–1.0 mg (HR < 60 bpm- Stop)
Short-acting B-blocker (esmolol/landiolol)	Esmolol: 80 mg for 15 to 30 s followed by 150 to 300 μg/kg/min maintenance dose based on need over 4–5 min—Landiolol: 0.1–0.3 mg/kg IV bolus or IV infusion 10–40 μg/kg/min as maintenance dose up to 24h in case of need.
Calcium channel blocker (verapamil/diltiazem)	0.075–0.15 mg/kg IV
Amiodarone	150 mg IV bolus IV infusion of 300 mg over 30 min

Bpm: beats per minute; HR: heart rate.

## Data Availability

The data presented in this study are available in [2021 ESC/EACTS Guidelines for the management of valvular heart disease] at [https://doi.org/10.1093/eurheartj/ehab395], accessed on 28 August 2021, and [2022 ESC Guidelines on cardiovascular assessment and management of patients undergoing non-cardiac surgery], at [https://doi.org/10.1093/eurheartj/ehac270], accessed on 26 August 2022, and [2020 ACC/AHA Guideline for the Management of Patients With Valvular Heart Disease], at [https://doi.org/10.1161/CIR.0000000000000923], accessed on 17 December 2020.
